# Effect of Diclofenac with B Vitamins on the Treatment of Acute Pain Originated by Lower-Limb Fracture and Surgery

**DOI:** 10.1155/2012/104782

**Published:** 2011-10-31

**Authors:** Héctor A. Ponce-Monter, Mario I. Ortiz, Alexis F. Garza-Hernández, Raúl Monroy-Maya, Marisela Soto-Ríos, Lourdes Carrillo-Alarcón, Gerardo Reyes-García, Eduardo Fernández-Martínez

**Affiliations:** ^1^Área Académica de Medicina del Instituto de Ciencias de la Salud, Universidad Autónoma del Estado de Hidalgo, 42090 Pachuca, HGO, Mexico; ^2^Research and Traumatology Departments, Hospital del Niño DIF, 42090 Pachuca, HGO, Mexico; ^3^Hospital General de los Servicios de Salud del Estado de Hidalgo, 42090 Pachuca, HGO, Mexico; ^4^SubDirección de Investigación de los Servicio de Salud de Hidalgo, 42030 Pachuca, HGO, Mexico; ^5^Sección de Estudios de Posgrado e Investigación, Escuela Superior de Medicina, IPN, 11340, DF, Mexico

## Abstract

The aim of this study was to compare the efficacy of diclofenac, for the treatment of acute pain originated by lower-limb fracture and surgery, with that of diclofenac plus B vitamins. This was a single-center, prospective, randomized, and double-blinded clinical trial. Patients with lower-limb closed fractures rated their pain on a 10 cm visual analog scale (VAS). Patients were then randomized to receive diclofenac or diclofenac plus B vitamins (thiamine, pyridoxine, and cyanocobalamin) intramuscularly twice daily. Patient evaluations of pain intensity were recorded throughout two periods: twenty-four hours presurgery and twenty-four hours postsurgical. One hundred twenty-two patients completed the study. The subjects' assessments of limb pain on the VAS showed a significant reduction from baseline values regardless of the treatment group. Diclofenac plus B vitamins combination was more effective to reduce the pain than diclofenac alone. The results showed that the addition of B vitamins to diclofenac increased its analgesic effect. The novelty of this paper consists in that diclofenac and diclofenac plus B vitamins were useful for treatment of acute pain originated by lower-limb fracture and surgery.

## 1. Introduction 

A number of situations are prone to develop pain symptomatology, such as tissue degeneration, infection, inflammation, cancer, trauma, surgery, and limb fractures. Each of these physiological abnormalities requires a therapeutic approach different from the last. In acute pain, caused by fracture and/or surgery, several classes of analgesics have been utilized. These basic remedies for analgesia, however, are still confined to a small number of medications, including nonsteroidal anti-inflammatory drugs (NSAIDs), local anesthetics, and opioids. In addition, most of these drugs have side effects, limiting their use in clinical practice [1, 2]. 

The clinical use of combinations of analgesic agents has increased significantly in the last few years. The purpose is to associate two or more drugs with different mechanisms of action, in hopes of achieving a synergistic interaction that yields a sufficient analgesic effect with low doses of each agent, therefore, reducing the intensity and incidence of untoward effects [3]. 

B vitamins are a water-soluble group of vitamins including thiamine, riboflavin, niacin and niacinamide, pyridoxine, cobalamin, folic acid, pantothenic acid, biotin, choline, inositol, and para-aminobenzoic acid (PABA). In particular, some of these B vitamins (thiamine, pyridoxine and cyanocobalamin) have been used, not only in the treatment of pain and inflammation resulting from vitamin deficiency but also alone or in combination with diclofenac or other NSAIDs for various painful diseases such as polyneuropathies, degenerative diseases of the spinal column, rheumatic diseases lumbago and pain originated from tonsillectomy [4–8]. However, most clinical studies have evaluated this combination in neuropathic pain [4–6, 8]. Recently, a study demonstrated the utility of the diclofenac-B vitamins combination in the pain originating from tonsillectomy surgery [7]. Nevertheless, the sample size of this last study was too small to be representative and the administration route was the intravenous via. On the other hand, this diclofenac-B vitamins combination has never been tested in the acute pain produced by fracture or other kind of surgical procedures. Therefore, the main objective of the present clinical study was to compare the efficacy and tolerability of diclofenac for treatment of acute pain following lower-limb fracture and surgery, with that of diclofenac in combination with B vitamins (thiamine, pyridoxine, and cyanocobalamin). Indeed, we previously conducted a pilot study with 14 patients, with the aim of establishing the adequate experimental conditions as well as to calculate the appropriate sample size, wherein both treatments were equally effective in reducing pain [9].

## 2. Materials and Methods

This was a single-center, prospective, randomized and double-blinded clinical trial. The study was carried out at the Hospital General SSH Pachuca, Hidalgo, Mexico from January 2008 to February 2010. The study protocol was approved by the Ethic and Investigation Committees from the Hospital General SSH Pachuca, Hidalgo, Mexico as well as this was conducted according to the Declaration of Helsinki.

The participants of the study were patients with lower-limb closed fractures, ranging in age from 18 to 55 years, with acute pain ≥5 cm according to a 10 cm visual analog scale (VAS; 0 = no pain and 10 = the worst pain), good health determined by clinical history and laboratory studies, without sanguineous dyscrasias or hypersensitivity to drugs to be employed, and who consented to participate voluntarily. 

After giving their consent, patients rated their pain on a VAS, and they were then randomized into one of two groups receiving 75 mg diclofenac or 75 mg diclofenac plus B vitamins (thiamine: 100 mg, pyridoxine: 100 mg, and cyanocobalamin: 1 mg) twice daily (all intramuscularly). Patient evaluations of pain intensity were recorded throughout two periods: twenty-four hours presurgical and twenty-four hours postsurgical. Twenty-four hours after the first drug administration, patients underwent elective lower-limb surgery. Standardized general anesthetic techniques were used for all patients. Patients received 50 mg ranitidine intravenously twice a day throughout the study. If the pain was not controlled after two hours, patients received rescue treatment with morphine. At the end of the study, the improvement in pain levels was evaluated by a Likert scale. The categorical Likert response alternatives consisted of four descriptions. Responses were rated 0–3: 0 = complete relief, 1 = moderate relief, 2 = slight relief, 3 = without relief. Gastrointestinal side effects, rash, or spontaneous complaints of other adverse effects such as postsurgical bleeding problems during the postoperative phase were registered. 

Data are shown as the mean ± SEM. Data were evaluated using *t* student and a nonparametric statistical analysis, the Mann-Whitney *U* test. *P* < 0.05 was required for significance.

## 3. Results 

One hundred twenty-two patients completed the study, sixty-two in the diclofenac group (forty-two male and twenty female) and sixty in the diclofenac with B vitamins group (twenty-seven male and thirty-three female). The mean ± standard deviation age in the diclofenac group was 37.9 ± 10.5 years and 35.0 ± 8.8 years in the diclofenac plus B vitamins group, which does not represent a significant difference between the groups (*P* > 0.05). 

In the study presented here, all patients received medication for forty-eight hours and the acute pain induced by lower-limb fracture and surgery was monitored and recorded. The lower-limb fractures that the patients presented with were 8 fractures of the patella, 47 ankle fractures, 24 tibia shaft fractures, 14 tibial plateau fractures, 20 diaphyseal fractures of the femur, 6 subtrochanteric femoral fractures, 2 fractures of the calcaneus, and 1 fracture of the talus. There was no statistically significant difference in the type of fracture and gender between the two treatment groups (*P* > 0.05).

**Figure 1:**
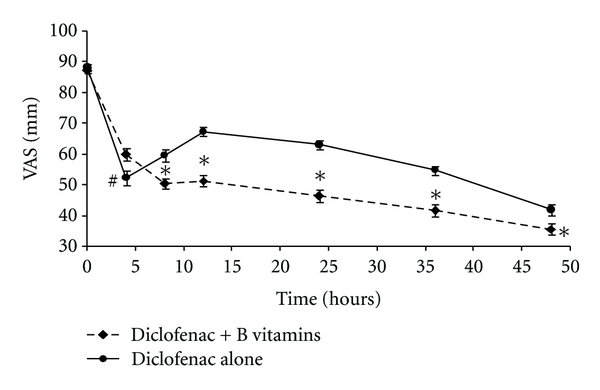
Time course of the effect of diclofenac alone or diclofenac plus B vitamins for the treatment of pain produced by lower-limb fracture and surgery. The points represent the average ± standard error of the media values of the visual analogous scale (VAS) evaluated at different hours. *Significantly different from diclofenac group (*P* < 0.05). ^#^Significantly different from diclofenac + B vitamins group (*P* < 0.05).

The subjects' assessment of limb pain on the VAS in both treatments showed a significant reduction from baseline at 4, 8, 12, 24, 36, and 48 hours after treatment. Diclofenac plus B vitamins was more effective to reduce the pain than diclofenac alone at 8, 12, 24, 36, and 48 hours after treatment (*P* < 0.05) (Figure 1). However, diclofenac was more successful to decrease the pain than diclofenac plus B vitamins at 4 hours after treatment (*P* < 0.05) (Figure 1). The value in the Likert scale of the diclofenac plus B vitamins group was 1.37 ± 0.5 with this value being statistically different at the value of 1.56 ± 0.5 for the diclofenac group. No statistical difference was found when comparing the groups according to gender and type of fracture (*P* > 0.05).

Rescue treatments were not applied. All the patients reported pain in the administration site, but generally speaking, all the regimens were well tolerated. None of the patients had any bleeding complication or gastrointestinal complain before or after surgery.

## 4. Discussion

Fractures of the lower limb are common, especially in the elderly, and are often associated with considerable morbidity and lengthy hospitalization. The immediate goal of treating acute lower-limb fractures is to decrease pain and swelling as well as to protect adjacent structures from further injury. For this reason, after immobilization of the lower limb, NSAIDs are invaluable in treating these musculoskeletal conditions, primarily due to their analgesic and anti-inflammatory effects. Unfortunately, NSAIDs propensity to cause gastrointestinal damage and patient discomfort limits their use. It is known that as many as two to four percent of patients who take NSAIDs during long-term therapy may have serious gastrointestinal side effects such as perforation, ulceration, or bleeding [10, 11]. An effective strategy to decrease the occurrence of these adverse effects is to combine an NSAID with two or more analgesics, each one with different mechanisms of action. The synergistic outcome achieved yields a sufficient analgesic effect with relatively low doses, reducing the intensity and incidence of untoward effects accordingly [3]. In this regard, it has been demonstrated that the combination of diclofenac and B vitamins is effective in relieving neuropathic pain [4, 5, 8]. Likewise, it is possible to reduce the diclofenac dosage and/or the duration of the treatment [4–6, 8]. A recent study showed that diclofenac plus B vitamins were not superior to diclofenac alone in the treatment of pain originated from tonsillectomy [7]. However, in this same study, the total dose of diclofenac was 45% less in the diclofenac plus B vitamins group suggesting a potential benefit to this approach to analgesic therapy. In a more recent report, it was demonstrated that after 3 days of treatment, a statistically significant higher proportion of subjects with lumbago who received diclofenac plus B vitamins completed the study due to the treatment success compared with patients that received diclofenac alone [8]. Furthermore, the combination therapy yielded superior results in pain reduction, improvement of mobility and functionality [8]. 

In the present study, both diclofenac and diclofenac plus B vitamins were able to produce an analgesic effect in patients with acute pain originating from lower-limb fracture and surgery. Likewise, at certain points of time the diclofenac-B vitamins combination provided a significantly greater analgesia than the single-agent diclofenac. In this last case, B vitamins potentiated the analgesia produced by diclofenac. There is evidences that B vitamins in their separate forms (B1, B6, and B12) have antinociceptive or analgesic effects. In this regard, in animal experiments, thiamine (B1 vitamin) was able to produce antinociception in the model of pain induced by acetic acid in mice, in the second phase of the formalin test in mice and in the model of pain induced by electrical stimulation of afferent fibers [12, 13]. Clinical studies showed that administration of a derivative of thiamine, benfothiamine, caused a significant improvement in patients with alcoholic polyneuropathy and in patients with diabetic neuropathy [14, 15]. On the other hand, pyridoxine (B6 vitamin) produced antinociception during the formalin test in mice and in the model of acetic acid in mice [12]. Clinical assays revealed that administration of pyridoxine improves symptoms that occur in the carpal tunnel syndrome and peripheral polyneuropathy [16, 17]. Finally, a clinical study demonstrated that systemic administration of cyanocobalamin (vitamin B12) to patients with low back pain was able to produce a significant decrease in pain and also decreased the consumption of acetaminophen as adjuvant therapy [18]. However, with the present experimental design it is impossible to know which B vitamin is the responsible of the better analgesia.

The increased analgesia with the diclofenac-B vitamins combination differs from the similar analgesic effects observed with diclofenac alone and diclofenac plus B vitamins in the treatment of acute postoperative pain after tonsillectomy [7]; probably, such a difference was due to the different kind of acute pain and the dissimilar administration pathway, since we used the intramuscular administration, while in that study the authors used intravenous infusion over a 12 h period [7]. To our knowledge, in our country there is no the pharmaceutical form of B vitamins for intravenous administration; there are only presentations for oral and intramuscular administration. A dosage form for intravenous administration would be very useful in cases where it cannot be used orally (e.g., in unconscious patients) or intramuscularly.

Diclofenac is an NSAID that exhibits potent analgesic and anti-inflammatory properties. It is known that diclofenac as well as other nonselective NSAIDs are able to impair prostaglandin synthesis by inhibiting the cyclooxygenase isozymes COX-1 and COX-2 in injured tissues and in the central nervous system. However, there is also evidence that diclofenac exhibits additional prostaglandin-independent properties that mediate its antinociceptive effects. For instance, diclofenac is able to inhibit H^+^-gated channels in sensory neurons, increase the concentration of kynurenate (an endogenous antagonist of NMDA receptors), stimulate the nitric oxide-cGMP-K^+^ channels pathway, and activate metformin- and phenformin-dependent mechanisms to induce antinociception [19–23]. On the other hand, several studies demonstrated antinociceptive and antihyperalgesic effects with the mixture of thiamine, pyridoxine, and cyanocobalamin in the models of hyperalgesia induced by carrageenan, in the pressure testing of the tail, and in the formalin model [24, 25]. Regarding the action mechanisms by which B vitamins produce their effects, it has been suggested that these result from the activation of several systems. For example, pyridoxine alone or in combination with thiamine and cyanocobalamin was able to increase the synthesis and secretion of serotonin in various brain regions [26, 27]. Furthermore, the analgesic effects of B vitamins have been associated with an increase in inhibitory control of afferent nociceptive neurons in the spinal cord [28] and reduced the response of thalamic neurons to nociceptive stimulation [29]. More recently, it was confirmed that the analgesic effects induced by B vitamins were partially blocked by naloxone, suggesting that B vitamins could release endogenous opioids that could activate opioid receptors [25]. Besides, there is experimental evidence suggesting that the effects induced by the combination of B vitamins involve the nitric oxide-cGMP system [30, 31]. However, other mechanisms have been proposed, for example, it has been shown that pyridoxine is capable of blocking the synthesis of prostaglandin E2 in humans [32]. In light of this evidence, it is possible to suggest that several mechanisms could be implicated in the diclofenac-B vitamins combination to obtain a major analgesia in comparison with the analgesia by diclofenac alone. The real mechanisms involved in the potentiation for the combination await future elucidation. 

Patients often experience unpredictable therapeutic and diagnostic procedural-related pain in emergency rooms that can be associated with considerable stress and anxiety [33]. Although procedural pain may be reduced by a variety of psychological and pharmacological interventions [33], most of these are not given in all the nonelective settings. For example, in our study, after the intramuscular injection all the patients reported pain at the administration site; however, the characteristics of this pain were not evaluated or recorded. 

One weakness of our study was that participants had different types of lower-limb fractures and surgeries, and this diversity could have affected the results in favor of providing a better analgesic effect with the combination of diclofenac with B vitamins. However, it was noted that although the patients had different types of fractures, there was no statistically significant difference in the level of pain that both groups of patients presented with on their admission. Therefore, it is necessary to evaluate the effectiveness of this combination in a particular type of fracture or surgery, either member. 

In conclusion, the present study gives evidence that the combination of diclofenac plus B vitamins could be a safe and inexpensive postsurgical analgesic strategy. Likewise, it is necessary to undertake controlled studies using this combination in different states of acute postsurgical pain to demonstrate its security and efficacy.
